# Quantification of 3-Dimensional Confluence-Atrial Morphology in Supracardiac Total Anomalous Pulmonary Venous Connection

**DOI:** 10.1016/j.jacasi.2024.05.002

**Published:** 2024-06-25

**Authors:** Guocheng Shi, Meiping Huang, Yuchen Pei, Peng Huang, Chen Wen, Jin Shentu, Hao Zhang, Zhongqun Zhu, Yumin Zhong, Lisheng Wang, Huiwen Chen

**Affiliations:** a Department of Cardiothoracic Surgery, Congenital Heart Center, Shanghai Children’s Medical Center, Shanghai Jiao Tong University School of Medicine, Shanghai, China; b Department of Catheterization Laboratory, Guangdong Cardiovascular Institute, Guangdong General Hospital, Guangdong, China; c Institute of Image Processing and Pattern Recognition, Department of Automation, Shanghai Jiao Tong University, Shanghai, China; d Department of Cardio-Thoracic Surgery, Hunan Children’s Hospital Changsha, China; e Department of Radiology, Shanghai Children’s Medical Center, Shanghai Jiao Tong University School of Medicine, Shanghai, China

**Keywords:** 3-dimensional computed tomography, congenital heart disease, pulmonary vein stenosis, total anomalous pulmonary venous connection

## Abstract

**Background:**

Pulmonary vein stenosis (PVS) continues to be a major complication after surgical repair of total anomalous pulmonary venous connection (TAPVC). Recent studies suggest that the morphology of pulmonary venous confluence and the left atrium (LA) is associated with PVS. However, there are limited data on the prognostic value of integrating quantitative confluence-atrial morphology into risk stratification.

**Objectives:**

This study sought to evaluate the prognostic impact of novel imaging metrics derived from 3-dimensional (3D) computed tomography angiography (CTA) modeling on postsurgical PVS (PPVS) in the supracardiac TAPVC (sTAPVC) setting.

**Methods:**

Patients undergoing sTAPVC repair in 2017 to 2022 from 3 centers were retrospectively reviewed. Study investigators developed 3D CTA modeled geometric features to quantify confluence-atrial morphology that were analyzed with regard to PPVS.

**Results:**

Of the 162 patients (median age 61 days; 55% having preoperative pulmonary venous obstruction [prePVO]) included, 47 (29%) with PPVS at a median of 1.5 months ([quartile 1-quartile 3: 1.5-3.0 months]). In the univariable analysis, the indexed total volume of the LA and confluence (iTVLC) and the ratio of the corresponding confluence length to the mean distance between the LA and confluence (CCL/mDBLC ratio) were significantly associated with PPVS. In a multivariable model adjusting for prePVO and age, the iTVLC and CCL/mDBLC ratio independently predicted PPVS (HR: 1.15; 95% CI: 1.06-1.25; and HR: 1.20; 95% CI: 1.08-1.35, respectively, all *P* < 0.01). Specifically, an iTVLC ≥20 cm^3^/m^2^ and a CCL/mDBLC ratio ≥7.7 were significantly associated with a reduced risk of PPVS.

**Conclusions:**

Quantification of 3D confluence-atrial morphology appears to offer a deeper and better metric to predict PPVS in patients with sTAPVC.

Direct anastomosis between the left atrium (LA) and the pulmonary venous confluence (PVC) is the standard of care procedure for supracardiac total anomalous pulmonary venous connection (sTAPVC), which is the most common subtype of total anomalous pulmonary venous connection (TAPVC) (∼40%).^1^, ^2^, ^3^ However, postsurgical pulmonary vein stenosis (PPVS) occurs in 11% to 17% of this subgroup and remains a treatment challenge, usually requiring multiple reinterventions and associated with substantial morbidity and mortality.^1^^,^^4^, ^5^, ^6^

Previous studies generated plausible explanations for PPVS that mechanotransduction in the pulmonary venous wall in response to flow alterations contributes to neointimal formation characterized by myofibroblast deposition.^7^, ^8^, ^9^ Anatomical alignment between the PVC and the LA is crucial for avoiding geometric distortion or stretch of the individual pulmonary veins (PVs), thereby optimizing the flow pattern and lowering the risk of PPVS. Conversely, achieving a large and patent atriovenous anastomosis free of PV distortion can be challenging because it is usually complicated by morphologic heterogeneity with respect to the confluence position, size, or shape, and left atrial size.^1^ Currently, morphologic assessment in this entity relies mostly on 2-dimensional (2D) echocardiography and computed tomography angiography (CTA) or cardiac magnetic resonance.^10^ However, plane angulation and slice selection may affect the cross-sectional 2D diameter measurements and contribute to interobserver or intraobserver variability. Additionally, the 2D measurements cannot fully describe the complex spatial information and geometric shape, thus creating an unmet need for a profound understanding of the involved 3-dimensional (3D) anatomical structure. Filling this knowledge gap may be an important step toward enhancing the understanding of the complex confluence-atrial morphology, which has been demonstrated to be closely related to PPVS.^11^, ^12^, ^13^

In this study of quantifying confluence-atrial positional relationship and geometric features by using 3D computed tomography (CT) modeling in the sTAPVC setting, we noticed the association between confluence-atrial morphology and PPVS. We hypothesized that this observation could be of clinical relevance.

## Methods

### Study design

We conducted a retrospective, observational study involving 3 tertiary hospitals in China (Shanghai Children’s Medical Center [SCMC], Guangdong General Hospital, and Hunan Children’s Hospital). SCMC served as the data coordinating center and received the study data transmitted from the other 2 clinical sites. Institutional Review Board approval was received for each site, and written informed consent was obtained from all participants’ guardians. This study complies with the Declaration of Helsinki.

### Patients and data collection

Patients undergoing surgical repair for sTAPVC between March 2017 and September 2022, with CTA performed shortly before surgery, were consecutively included. Participants from SCMC formed the derivation cohort, and participants from the other 2 hospitals formed the external validation cohort. Exclusion criteria were as follows: 1) patients aged >18 years; 2) patients having TAPVC in association with heterotaxy, atrial isomerism, or other cardiac anomalies except atrial septal defect (ASD) or patent ductus arteriosus; 3) patients without available preoperative CTA scans or with inadequate CTA image quality to allow reliable morphologic analysis; and 4) patients who did not undergo atriovenous anastomosis.

Data collection included the following: patient baseline demographics (age at surgery, sex, body surface area [BSA], oxygen saturation); preoperative and surveillance imaging information, including echocardiographic and CTA data; clinical information on requirement of preoperative treatment (mechanical ventilation, infusion therapy); time of operation (emergency [≤24 hours on presentation] vs nonemergency surgery [>24 hours on presentation]); procedural details; postoperative course during hospitalization; and follow-up status. Data were collected from the electronic medical records and were analyzed retrospectively. The echocardiographic data were reread and reanalyzed centrally in the echocardiographic core laboratory of SCMC. Preoperative pulmonary venous obstruction (prePVO) was defined on the basis of the combined evaluation of oxygen saturation (<90% at rest), echocardiographic reports (nonphasic Doppler velocity of >1.8 m/s within the pulmonary venous draining course and/or restrictive ASD^11^), and/or CTA findings (minimal diameter/reference vessel diameter <50%^14^). Specifically, Doppler velocity of >1.8 m/s has been consistently used as the diagnostic criterion of pulmonary vein stenosis (PVS) since our center established the TAPVC program in 2016.^3^^,^^4^^,^^11^^,^^12^

### CTA imaging acquisition and analysis

The CTA data (full DICOM [Digital Imaging and Communications in Medicine] data sets) were transmitted to the core laboratory of SCMC. For the purpose of this study, the CTA data were reread and reanalyzed by a single expert investigator (Y.Z.) with >20 years of experience in pediatric cardiac imaging who was blinded to the follow-up data.

#### Segmentation and 3D modeling

**Figure 1:**
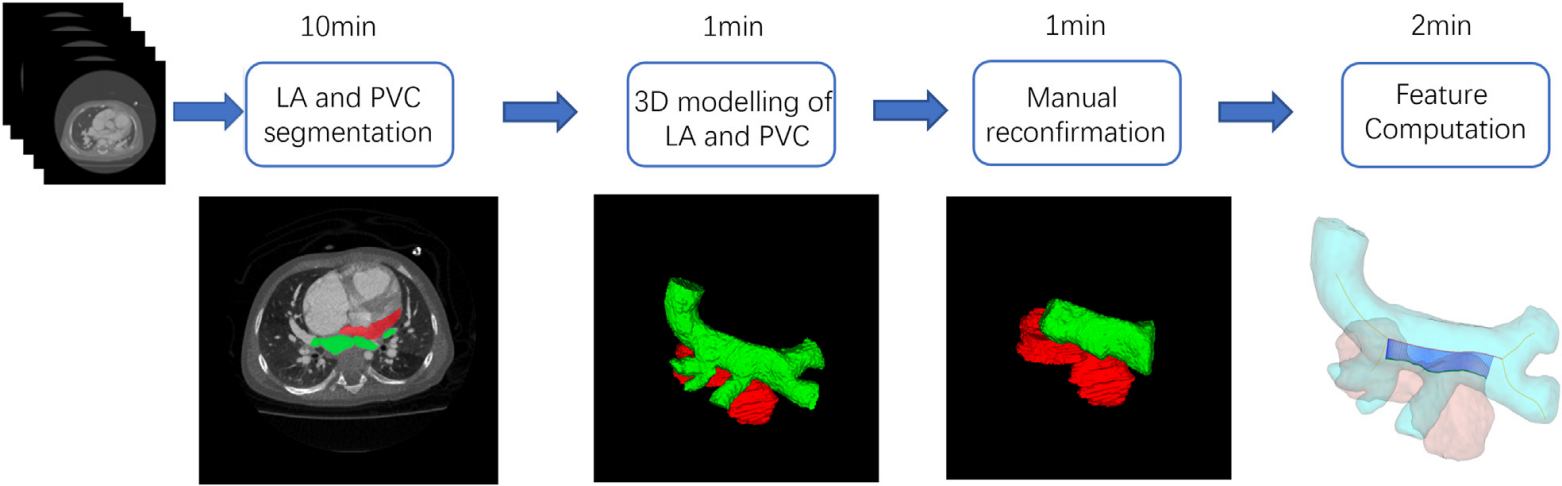
Study Workflow Representative overview of the work process including semiautomated segmentation from the multidetector computed tomography images, a 3-dimensional (3D) computational model of the left atrium (LA) and pulmonary venous confluence (PVC), and quantification of the morphologic features.

Details of the process were elaborated in our previous publication.^15^ Briefly, a convolutional neural network was used to segment the PVC and LA automatically from 3D CTA. We incorporated the attention mechanism^16^ into the V-Net^17^ by implementing the attention mechanism in spatial and channel attention blocks, which can guide the network to focus on the important spatial position and feature channels to extract more useful features for better segmentation performance. Thereafter, the expert investigator (Z.Y.M.) performed the manual revision to ensure the accuracy of the segmentation. Figure 1 shows the workflow of this process.

#### Quantification of the morphologic features

**Figure 2:**
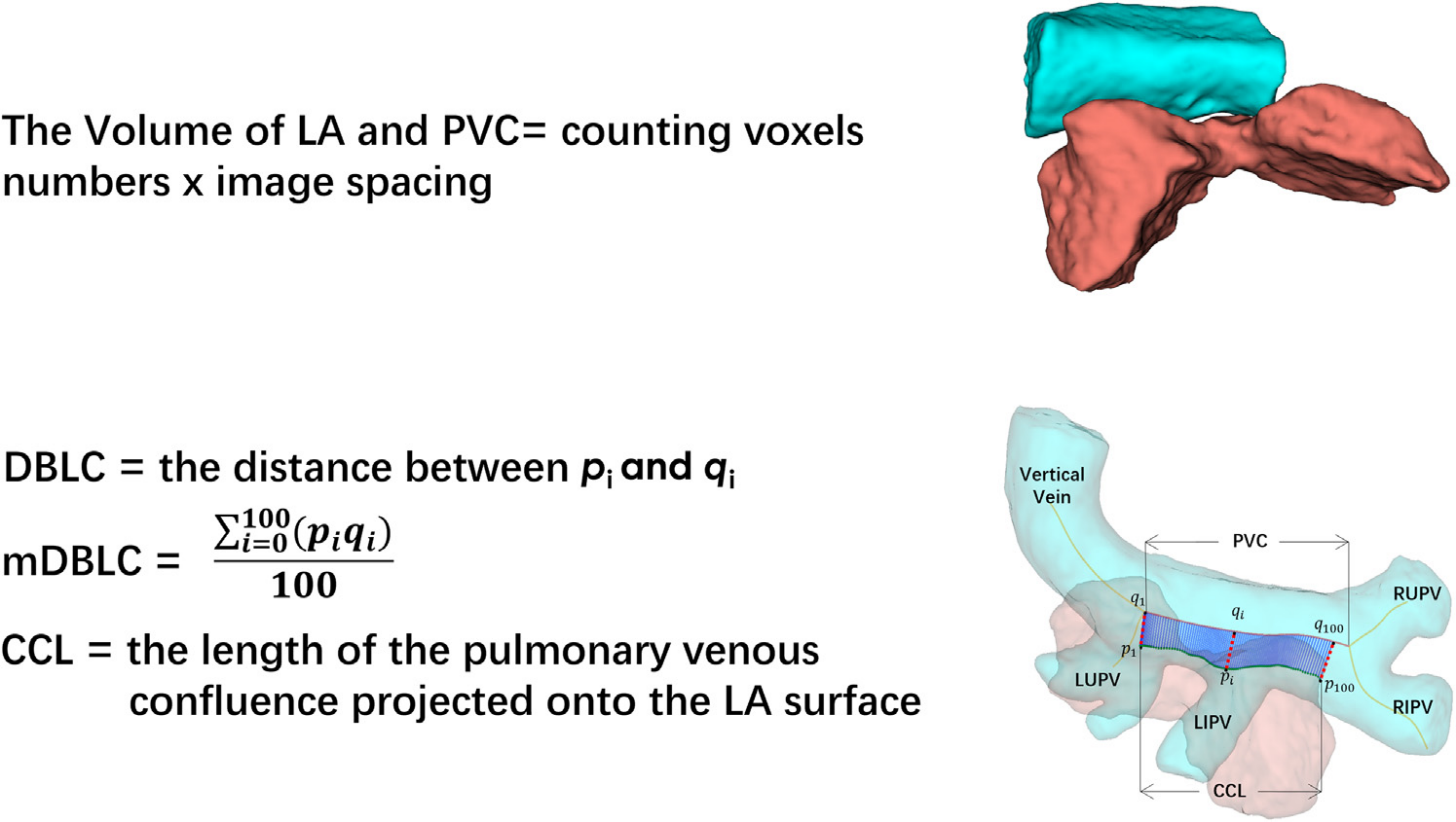
Quantification of 3-Dimensional Computed Tomography Angiography Modeled Geometric Features (A) Volume measurements of the left atrium (LA) and pulmonary venous confluence (PVC). (B) The positional relationship between the left atrium and the pulmonary venous confluence can be reflected by the distance between the left atrium and the confluence (DBLC) and the corresponding confluence length (CCL). Furthermore, the CCL/mDBLC ratio is used as an integrated indictor. mDBLC = mean distance between the left atrium and the confluence; LIPV = left inferior pulmonary vein; LUPV= left upper pulmonary vein; RIPV = right inferior pulmonary vein; RUPV = left upper pulmonary vein.

The centerline paths of the confluence, vertical vein (VV), and individual PVs were computed automatically using flux-driven centerline extraction.^18^ The confluence was marked from the plane where the right upper and inferior PVs joined the plane where the VV and left upper PV joined (Figure 2). The confluence was projected onto the left atrial surface to obtain the corresponding confluence on the basis of the stereo geometry;^15^^,^^19^ thereafter, the corresponding confluence length (CCL) could be determined. The corresponding confluence was discretely sampled at 100 points (p_*i*_, *I* = 1….100) at equal intervals. Accordingly, 100 corresponding projection points (q_*i*_, *I* = 1….100) in the centerline of the confluence could be identified for each sampling point (p_*i*_). The projection distance between the LA and the confluence (DBLC) could be measured, defined as the distance between p_*i*_ and q_*i*_, and therefore the mean DBLC (mDBLC) could be calculated (Figure 2). Volume measurements of the confluence and LA were estimated by counting voxels in their segmentation areas (Figure 2).

### Follow-up and outcome assessment

The primary endpoint was PPVS. The diagnosis of PPVS was mainly established on the basis of the following echocardiographic and/or CTA information: 1) nonphasic and continuous wave form with a velocity of >1.8 m/s in the confluence and/or individual PVs demonstrated by Doppler flow; and 2) ≥50% luminal narrowing in the involved PVs. In patients who developed PPVS, the condition was first detected by surveillance echocardiography; thereafter, CTA was required for further evaluation of the degree of stenosis at the left atrial junction or upstream. Patients’ surveillance echocardiographic data, as well as CTA if available, were reread by a senior pediatric cardiac specialist (Z.Z.) from SCMC. PPVS was then ascertained, and it could be further subcategorized into clinically evident and subclinical PPVS (patients who were asymptomatic, albeit having increased PV velocity noted on echocardiography).

Patients were required to be followed up through clinical visits at 1-, 3-, 6-, 9-, and 12-month intervals after the index TAPVC surgery and then annually thereafter. Physical examination and surveillance echocardiography with or without CTA were routinely required at each follow-up visit. Meanwhile, the senior physicians at each participating hospital would check on patients’ general status, respiratory issues (recurrent respiratory infection, dyspnea, cyanosis), and feeding intolerance to monitor the occurrence of PPVS. If there was a suspicion of the onset of PPVS, the patients were required to come to our hospitals for a timely visit irrespective of the scheduled time intervals. Follow-up time started on the date of the initial surgery and ended at the time of PPVS or September 30, 2022, whichever occurred first. Data from patients who were lost to follow-up were censored at the last day that their vital status was known.

### Statistical analysis

All data were analyzed with R software version 3.6.1 (R Foundation). A 2-tailed *P* value <0.05 was considered statistically significant. The normality of quantitative variables was determined using the Kolmogorov-Smirnov test. Continuous variables are expressed as median (quartile 1-quartile 3 [Q1-Q3]), and categorical variables are expressed in frequency and percentage. We used the Mann-Whitney *U* or Kruskal-Wallis test and the chi-square or Fisher exact test to estimate the statistical significance of continuous and categorical variable distributions between the study groups of interest. The Pearson correlation coefficient was used to determine the correlation between the morphologic parameters (BSA-adjusted total volume of LA and confluence [iTVLC] and CCL/mDBLC ratio) and aortic cross-clamping time. The Cox proportional hazards model was used to explore the association between PPVS and imaging metrics, as well as the clinical factors, and the results were expressed as HRs with 95% CIs. The Schoenfeld residuals test was used to validate the proportional hazard assumption for all variables, which was satisfied for the outcome of freedom from PPVS from the initial operation to the last follow-up. Variables with *P* < 0.05 in the univariable analysis were entered in multivariable models with a backward selection procedure. We relied on the events according to variable criteria and assessed the association between PPVS and morphologic parameters after adjusting for prePVO and age on basis of their known clinical relevance with PPVS. In addition, we used a restricted spline curve with 4 knots at the 5th, 35th, 65th, and 95th percentiles of the iTVLC and the CCL/mDBLC ratio to investigate further the relationship between the 2 imaging metrics and the HR change for PPVS. A competing risk analysis was not used because all deaths occurred secondary to PPVS. body surface area–adjusted volume of the pulmonary venous confluence [iPVC_volume_], body surface area–adjusted volume of left atrium [iLA_volume_].

A time-dependent area under the receiver-operating characteristic (ROC) curve^20^ was performed to measure the predictive performances of prePVO and the morphologic features (iPVC_volume_ [BSA-adjusted volume of the PVC], iLA_volume_ [BSA-adjusted volume of the LA], iTVLC, CCL/mDBLC ratio, and combination of iTVLC and CCL/mDBLC ratio) for PPVS during the follow-up period. Considering that the highest hazard for PPVS is usually observed within or around postoperative 1 year, this time point was additionally used to develop the ROC curve. Freedom from PPVS was estimated using the Kaplan-Meier method and was compared using the log-rank test.

## Results

### Study Group

In total, 162 patients (median age, 61 days; 62.3% male) were included. There were 102 and 60 patients in the derivation and validation cohorts, respectively. Patient demographics, including age, BSA, prePVO, oxygen saturation, and need for emergency operation or preoperative management, were comparable between the 2 cohorts (Table 1). Longer cardiopulmonary bypass time and duration of hospital stay were found in the derivation cohort, probably reflecting the intuitional variations in management protocols.

**Table 1:** Patient Baseline Characteristics, Intraoperative and Postoperative Data, and CTA-Derived Morphologic Parameters

	All Patients (N = 162)	Derivation Cohort (n = 102)	Validation Cohort (n = 60)	*P* Value
Baseline clinical characteristics				
Age, d	61.0 (30.8-150.5)	58.0 (30.0-148.3)	65.5 (36.3-204.0)	0.217
Male	101 (62.3)	61 (59.8)	40 (66.7)	0.406
BSA, m^2^	0.26 (0.22-0.32)	0.25 (0.22-0.32)	0.26 (0.22-0.34)	0.275
Preoperative PVO	55 (34.0)	31 (30.4)	24 (40.0)	0.212
Spo_2_, %	87.0 (80.0-91.3)	87.0 (80.0-92.0)	87.0 (78.0-90.8)	0.516
Preoperative treatment	60 (37.0)	39 (38.2)	21 (35.0)	0.738
Time of operation				0.459
Emergency	41 (25.3)	28 (27.5)	13 (21.7)	
Nonemergency	121 (74.7)	74 (72.5)	47 (78.3)	
Subtype of sTAPVC				0.094
Type Ia (left-sided VV)	146 (90)	95 (93)	51 (85)	
Type Ib (right-sided VV)	16 (10)	7 (7)	9 (15)	
Associated cardiac anomalies				
Patent ductus arteriosus	7 (4.3)	5 (4.9)	2 (3.3)	1.000
Atrial septal defect	162 (100)	102 (100)	60 (100)	*NS*
Intraoperative and postoperative data				
CPB time, min	92.0 (76.0-112.3)	97.5 (82.8-119.0)	80.0 (72.0-99.3)	<0.001
Aortic cross-clamping time, min	45.0 (36.8-58.0)	45.0 (34.8-58.0)	44.5 (38.0-57.8)	0.518
CCU stay, d	6.0 (4.0-8.0)	6.0 (4.0-8.0)	5.0 (4.0-7.8)	0.257
Hospital stay, d	14.0 (10.0-20.0)	16.0 (11.0-20.0)	10.0 (9.0-19.5)	0.007
Duration of surveillance, mo	24.0 (6.0-42.0)	24.0 (5.4-42.0)	24.5 (6.1-41.0)	0.958
Death	6 (3.7)	4 (3.9)	2 (3.3)	1.000
PPVS	47 (29.0)	30 (29.4)	17 (28.3)	1.000
CTA-derived morphologic parameters				
iPVC_volume_, cm^3^/m^2^	4.9 (3.0-7.6)	4.8 (3.1-7.1)	5.3 (3.0-8.0)	0.795
iLA_volume_, cm^3^/m^2^	14.4 (11.0-18.5)	14.3 (11.8-18.4)	14.4 (10.4-18.7)	0.846
iTVLC, cm^3^/m^2^	20.0 (15.7-24.8)	20.1 (16.5-24.0)	19.0 (14.1-25.5)	0.652
CCL, mm	11.4 (8.3-15.8)	11.6 (8.4-16.2)	10.9 (7.9-15.2)	0.278
mDBLC, mm	1.5 (1.3-1.8)	1.4 (1.2-1.8)	1.5 (1.3-1.8)	0.162
CCL/mDBLC ratio	7.6 (4.9-11.5)	8.4 (5.0-14.0)	6.7 (4.2-10.2)	0.065

Values are median (quartile 1-quartile 3) or n (%).

BSA = body surface area; CCL = corresponding confluence length; CCU = cardiac care unit; CPB = cardiopulmonary bypass; CTA = computed tomography angiography; iLA_volume_ = volume of left atrium after indexing to BSA; iPVC_volume_ = volume of pulmonary venous confluence after indexing to BSA; iTVLC = BSA-adjusted total volume of the left atrium and confluence; mDBLC = mean distance between left atrium and pulmonary venous confluence;; PPVS = postsurgical pulmonary vein stenosis; PVO = pulmonary venous obstruction; Spo_2_ = percutaneous arterial oxygen saturation; sTAPVC = supracardiac total anomalous pulmonary venous connection; VV = vertical vein.

Patients in the derivation cohort had a similar duration of follow-up compared with patients in the validation cohort (median, 24.0 months [Q1-Q3: 5.4-42.0 months] vs median 24.5 months [Q1-Q3: 6.1-41.0 months]; *P* = 0.96). Overall, PPVS occurred in 47 of the 162 cases (29%) at a median of 1.5 months (Q1-Q3: 1.5-3.0 months), including 30 of the 102 cases (29.4%) at a median of 1.5 months (Q1-Q3: 1.5-4.6 months) in the derivation cohort and 17 of 60 (28.3%) at a median of 1.5 months (Q1-Q3: 1.4-1.8 months) in the validation cohort. Of the 47 patients, 6 died of PPVS, 25 had subclinical PPVS and underwent ongoing close surveillance, and the remaining 16 patients had relief of restenosis following reoperation at a median of 1.5 months (Q1-Q3: 0.5-3.5 months) after the initial surgery. No death occurred in those patients who had no PPVS.

### Novel CTA-derived confluence-atrial morphologic parameters

In the entire cohort, compared with patients without prePVO, patients with prePVO had a smaller iTVLC (16.96 cm^3^/m^2^ [Q1-Q3: 13.26-20.45 cm^3^/m^2^] vs 21.80 cm^3^/m^2^ [Q1-Q3: 17.15-26.89 cm^3^/m^2^]; *P* < 0.001), BSA-adjusted LA volume (iLA_volume_: 13.15 cm^3^/m^2^ [Q1-Q3: 10.25-15.90 cm^3^/m^2^] vs 15.47 cm^3^/m^2^ [Q1-Q3: 12.32-19.38 cm^3^/m^2^]; *P* = 0.001), BSA-adjusted PVC volume (iPVC_volume_: 3.61 cm^3^/m^2^ [Q1-Q3: 2.62-4.95 cm^3^/m^2^] vs 6.20 cm^3^/m^2^ [Q1-Q3: 4.15-8.67 cm^3^/m^2^]; *P* = 0.001), and CCL/mDBLC ratio (6.16 [Q1-Q3: 4.45-10.29] vs 8.86 [Q1-Q3: 6.14-13.64]; *P* = 0.001). No differences in the iTVLC and CCL/DBLC ratio were observed in patients who had right VV vs left VV (iTVLC: 20.10 cm^3^/m^2^ [Q1-Q3: 16.10-25.43 cm^3^/m^2^] vs 18.84 cm^3^/m^2^ [Q1-Q3:13.92-22.13 cm^3^/m^2^]; *P* = 0.20; CCL/mDBLC ratio: 7.81 [Q1-Q3:4.92-12.14] vs 7.39 [Q1-Q3: 4.76-10.47]; *P* = 0.56). The distributions of the iTVLC and the CCL/mDBLC ratio were similar between the derivation and validation cohorts (Table 1).

Specifically, when stratified by age (neonates [0-30 days; n = 36], infants [1-12 months; n = 110], and children [>12 months; n = 16]) in the full sample, younger patients tended to have statistically smaller volumes of the LA, PVC, and total volume of left atrium and confluence (TVLC), even after adjustment for the patients’ BSA (Supplemental Table 1). In addition, younger patients had shorter DBLC (*P* = 0.042) and CCL (*P* = 0.009); however, no statistical difference of the CCL/DBLC ratio was observed among the 3 age subgroups (*P*=0.59) (Supplemental Table 1).

### Derivation cohort

**Figure 3:**
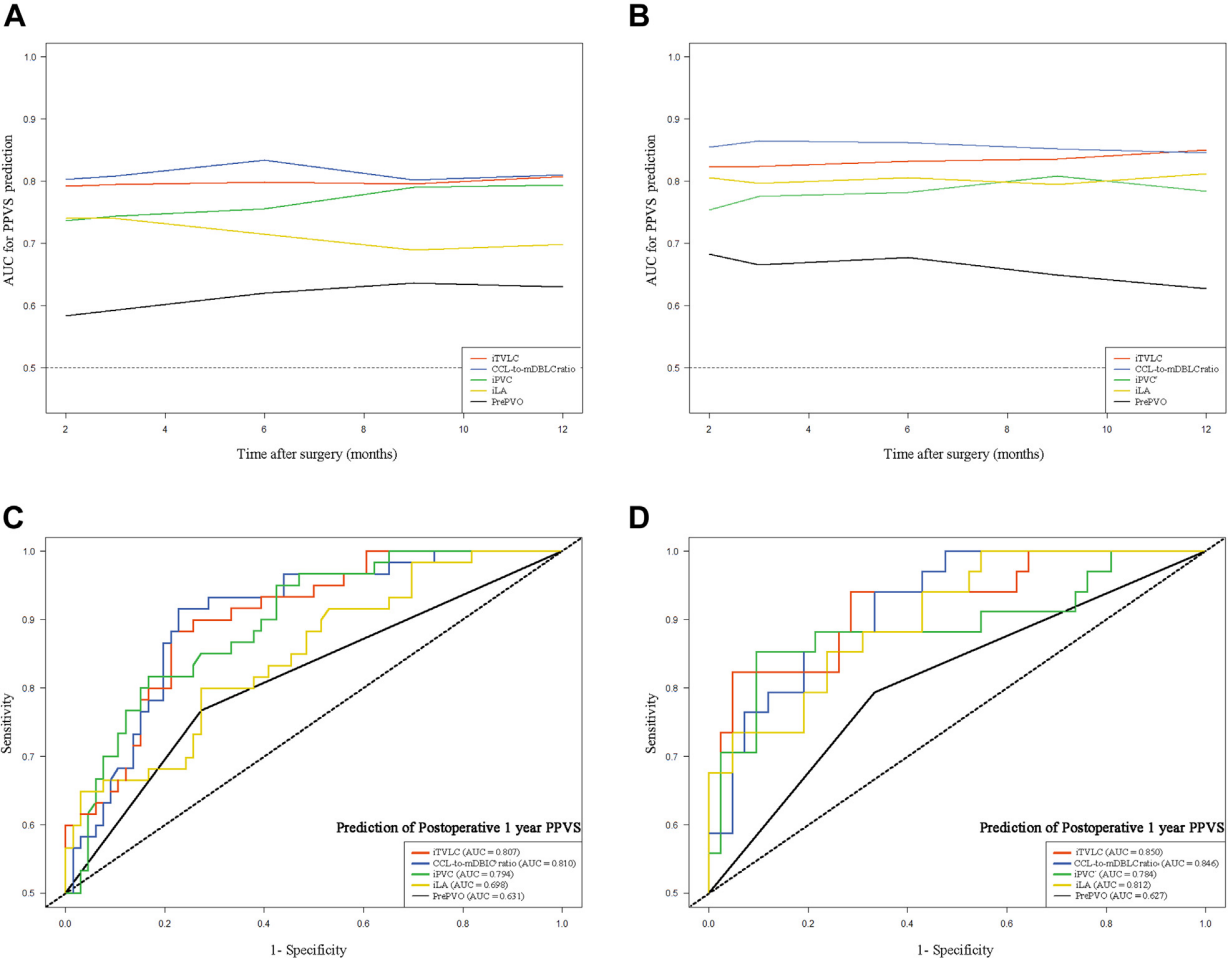
Time-Dependent Receiver-Operating Characteristic Analysis of PPVS Time-dependent area under the curve (AUC) analysis of the imaging metrics (body surface area–adjusted total volume of the left atrium and confluence [iTVLC], body surface area–adjusted volume of the pulmonary venous confluence [iPVC_volume_], body surface area–adjusted volume of left atrium [iLA_volume_], ratio of the corresponding confluence length [CCL] to the mean distance between the left atrium and the confluence [mDBLC]) and the clinical risk factor (preoperative pulmonary venous obstruction [prePVO]) in postsurgical pulmonary vein stenosis (PPVS) prediction in the (A) derivation cohort and (B) validation cohort, respectively. Comparison of the postsurgical pulmonary vein stenosis predictive power of the imaging metrics and preoperative pulmonary venous obstruction at postoperative 1 year in the (C) derivation cohort and (D) validation cohort.

We assessed the associations of CTA-derived morphologic parameters with PPVS after adjusting for patient age and prePVO (Table 2). The time-dependent ROC curves revealed that the discrimination of each CTA-derived morphologic parameter outperformed prePVO in predicting PPVS (Figure 3A). Interestingly, iTVLC showed better prognostic value than iPVC_volume_ or iLA_volume_ alone. Specifically in these analyses, the ROC AUCs for the iTVLC and CCL/mDBLC ratio were 0.807 (95% CI: 0.725-0.901) and 0.810 (95% CI: 0.721-0.899), respectively, for predicting postoperative 1-year PVS (Figure 3C). In addition, a smaller iTVLC (Pearson’s *r* = −0.34; *P* = 0.001) and CCL/mDBLC ratio (Pearson’s *r* = −0.26; *P* = 0.009) had strong correlation with increased aortic-cross clamping time.

**Table 2:** Associations Between the CTA-Derived Metrics and Postsurgical Pulmonary Vein Stenosis in the Derivation Cohort

CTA-Derived Morphologic Parameter	Unadjusted			Adjusted		
	*P* Value	HR	95% CI	*P* Value	HR	95% CI
iPVC_volume_, cm^3^/m^2^	<0.001	1.46	1.21-1.74	0.003^a^	1.39	1.12-1.70
iLA_volume_, cm^3^/m^2^	0.003	1.15	1.05-1.26	0.025^a^	1.11	1.03-1.22
iTVLC, cm^3^/m^2^	<0.001	1.17	1.09-1.26	0.001^a^	1.15	1.06-1.25
CCL, mm	<0.001	1.16	1.07-1.26	0.008^a^	1.13	1.03-1.22
mDBLC, mm	0.087	1.24	0.97-1.58	0.079^a^	1.26	0.97-1.63
CCL/mDBLC ratio	<0.001	1.24	1.16-1.35	0.001^a^	1.20	1.08-1.32

Abbreviations as in Table 1.

a After adjustment for preoperative pulmonary venous obstruction and patient age.

### Validation cohort

We confirmed in the validation cohort that all of the CTA-derived morphologic variables showed better discriminatory performance than prePVO in predicting PPVS (Figure 3B). The AUCs for the iTVLC and CCL/mDBLC ratio were 0.850 (95% CI: 0.758-0.971) and 0.846 (95% CI: 0.688-0.921), respectively, for predicting postoperative 1-year PVS (Figure 3D). Moreover, correlations between the morphologic parameters and increased aortic-cross clamping time (iTVLC: *r* = −0.45; *P* < 0.001; CCL/DBLC ratio: *r* = −0.34; *P* = 0.008) could be observed. Considering that there were only 17 PPVS events in the validation cohort, to avoid overfitting, we assessed the association between the imaging variables (iTVLC and CCL/mDBLC ratio) and PPVS after adjusting for patient age or prePVO, respectively; these 2 parameters were consistently associated with PPVS (Supplemental Table 2).

### Outcome analyses in the combined cohort

We combined the derivation and validation cohorts to increase the sample size (N = 162) further, to facilitate the follow-up analyses. In the univariate analysis for the entire cohort (total number of PPVS cases: 47), baseline clinical risk factors of PPVS included prePVO (HR: 2.54; 95% CI: 1.43-4.50; *P* = 0.002), patient BSA (HR: 3.52; 95% CI: 1.91-8.72; *P* < 0.001), emergency operation (HR: 2.05; 95% CI: 1.14-3.69; *P* = 0.017), and age (HR: 1.01; 95% CI: 1.00-1.02; *P* = 0.003). Only prePVO (HR: 1.91; 95% CI: 1.04-3.53; *P* = 0.038) was independently associated with PPVS in the multivariate analyses. We assessed the association between the CTA metrics (iTVLC, CCL/mDBLC ratio) after adjusting prePVO, and these 2 imaging parameters remained associated with PPVS (iTVLC: HR: 1.15; 95% CI: 1.08-1.22; *P* < 0.001; CCL/mDBLC ratio: HR: 1.26; 95% CI: 1.14-1.40; *P* < 0.001). In addition, integration of the 2 morphologic parameters outperformed prePVO in predicting PPVS (Supplemental Figure 1).

**Figure 4:**
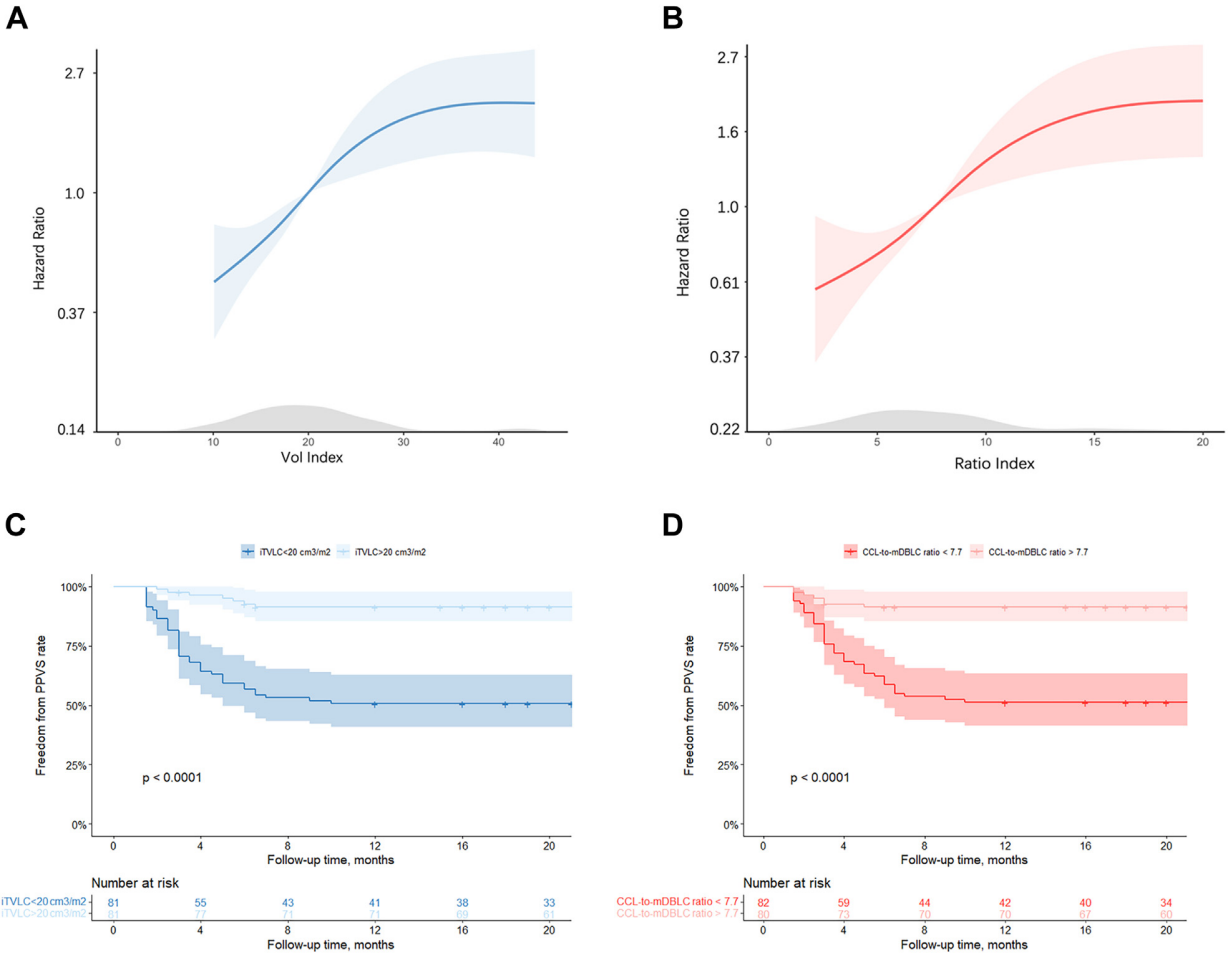
PPVS Analysis According to the Cutoff Value of Imaging Metrics The spline curves show the HR change for freedom from PPVS with 95% CIs across a range of values of (A) body surface area–adjusted total volume of the left atrium and confluence (iTVLC) values and (B) ratios of the corresponding confluence length (CCL) to the mean distance between the left atrium and the pulmonary venous confluence (mDBLC). The density plots beneath the curves show the distribution of the patients according to values of the body surface area–adjusted total volume of the left atrium and confluence and the CCL/mDBLC ratio. The Kaplan-Meier curves show that the patients with (C) a body surface area–adjusted total volume of the left atrium and confluence ≥20 cm^3^/m^2^ or (D) a CCL/mDBLC ratio ≥7.7 have a lower risk of postsurgical pulmonary vein stenosis (PPVS). Vol = volume.

**Figure 5:**
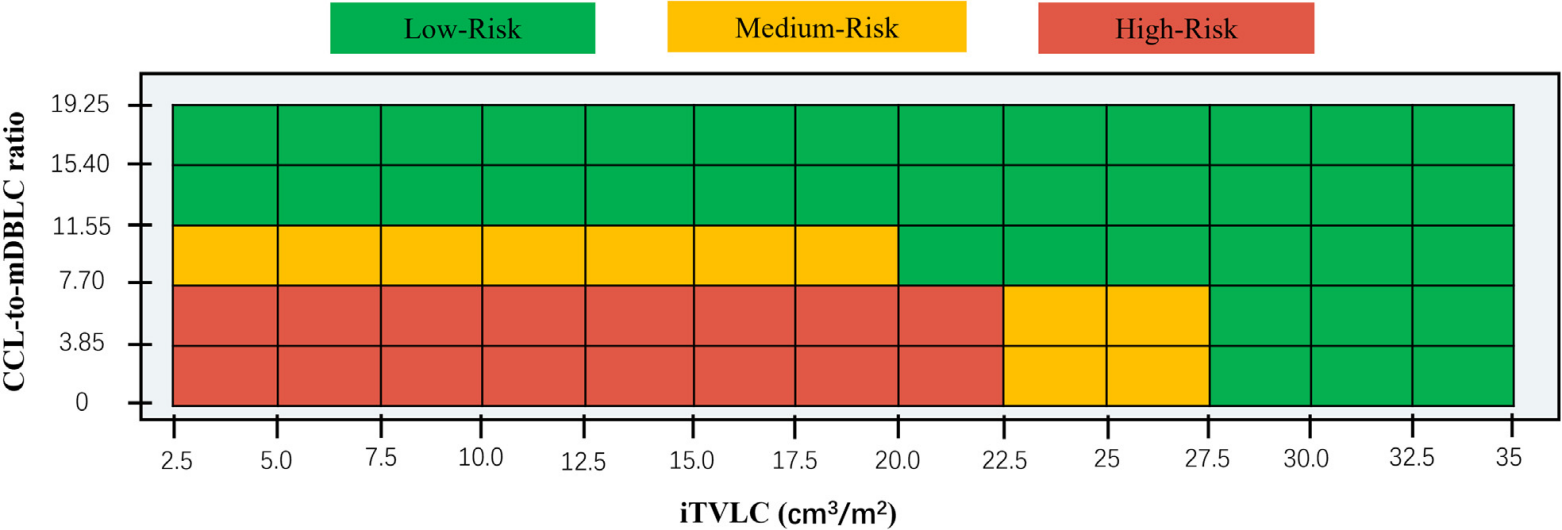
Risk Stratification on the Basis of the Imaging Metrics On the basis of the body surface area–adjusted total volume of the left atrium and confluence (iTVLC) and the ratio of the corresponding confluence length (CCL) to the mean distance between the left atrium and the pulmonary venous confluence (mDBLC), patients can be subcategorized as those having low (green), medium (orange), and high (red) risks of postsurgical pulmonary vein stenosis.

Spline curve analysis was performed, and the threshold values of the iTVLC (20.0 cm^3^/m^2^) and CCL/mDBLC ratio (7.7) were respectively identified to dichotomize the study group (Figures 4A and 4B). To verify the clinical relevance of spline curve findings, freedom from PPVS was analyzed according to the observed thresholds. Using the 20 cm^3^/m^2^ cutoff for the iTVLC, a markedly higher PPVS incidence was observed in small (<20 cm^3^/m^2^) vs large (≥20 cm^3^/m^2^) iTVLCs (*P* < 0.001) (Figure 4C). Similarly, PPVS incidence was increased in patients with a small (<7.7) vs large (≥7.7) CCL/mDBLC ratio (*P* < 0.001) (Figure 4D). Furthermore, we categorized those patients who had an iTVLC >20 cm^3^/m^2^ or a CCL/mDBLC ratio >7.7 into a low-risk group if there was no observed PPVS; otherwise, the patients were considered to be at moderate risk of PPVS (Figure 5).

## Discussion

This study demonstrates the critical concept for quantitative 3D morphologic analysis in sTAPVC. The key findings (Central Illustration) are as follows. First, the iTVLC and CCL/mDBLC ratio are 2 useful 3D CTA-derived quantitative morphologic features to identify patients at high risk of PVS after sTAPVC repair. Moreover, integration of these 2 parameters outperformed the conventional clinical factor (prePVO) in PPVS prediction. Second, a small iTVLC and CCL/mDBLC ratio reflected unfavorable morphology that appeared to increase procedural complexity and was associated with longer aortic cross-clamping times.

**Central Illustration:**
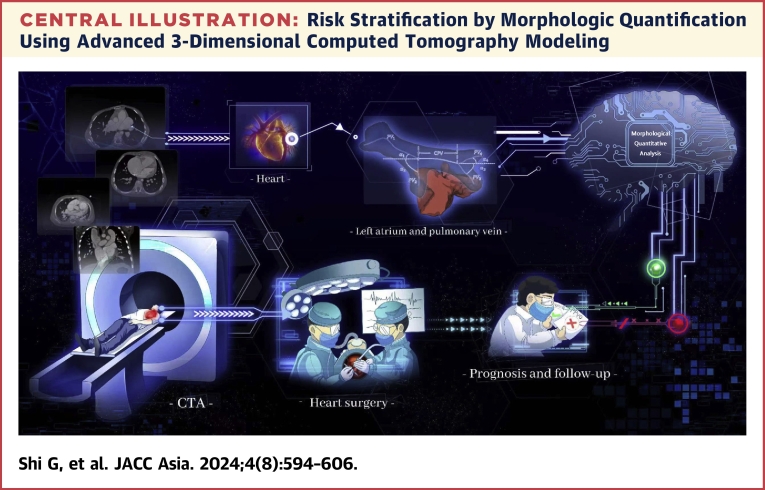
Risk Stratification by Morphologic Quantification Using Advanced 3-Dimensional Computed Tomography Modeling Morphologic quantification derived from advanced 3-dimensional computed tomography modeling assists in individualized risk stratification. CTA = computed tomography angiography.

Emerging evidence suggests neointima formation with myofibroblast-like cell deposition as a main histopathological change in PPVS wherein mechanotransduction in the cells of vessel walls in response to locoregional hemodynamic factors potentially plays a vital role.^7^^,^^8^^,^^21^^,^^22^ Unfavorable atriovenous anastomosis poses potent physical stimuli for venous wall cells, by driving the onset of vascular remodeling and consequently causing luminal stenosis. Previous study suggested that the tremendous morphologic heterogeneity in the setting of TAPVC increases procedural complexity.^1^ Unfavorable morphology results in anastomotic imperfection, thereby contributing to PPVS development. This finding highlights the need for identifying high-risk morphologic phenotypes. Unfortunately, studies focusing on this issue are limited, and most have been qualitative studies.^1^^,^^13^ The present work bridges this knowledge gap by analyzing the geometric features in sTAPVC on the basis of 3D CT modeling, in the hope of selecting patients at increased risk of PPVS.

We took the sum of volumes of confluence and LA as an indicator and found that it showed better prognostic value for predicting PPVS than LA or PVC volume alone after adjusting for patients’ BSA. Additionally, our data indicated that a smaller iTVLC had a strong association with prePVO. These findings merit special discussion. First, a study demonstrated that patients with obstructed pulmonary venous return had decreased left atrial reservoir and contractile functions.^23^ Moreover, such impairment in left atrial function exists even after TAPVC repair even though the small left side of the heart can increase postoperatively.^24^ There is a possibility that the mismatch reservoir function of the LA exposes the PV system to a high filling pressure postoperatively and drives the onset of vascular remodeling.^25^ This speculation is partially supported by the pathologic findings in the left atrial stenosis rat model, which revealed a progression of stenosis extending to the upstream PVs characterized by intimal fibrosis and medial hypertrophy, closely resembling the pathophysiology of PPVS.^26^ Second, there are some suggestions that a small confluence and LA add difficulty in achieving a large and patent atriovenous anastomosis that has a strong association with PPVS.^1^^,^^27^

Volumetric measurement of the LA and PVC is necessary but not sufficient because reconstruction of the anomalous PV drainage to the LA requires comprehensive consideration of not only the involved structures per se but also their positional relationships. We used mDBLC and CCL to quantify the spatial relationship between the confluence and the LA. Further, we uniquely integrated these 2 parameters and used the CCL/mDBLC ratio as an indicator, and we observed that a smaller CCL/mDBLC ratio was associated with an increased risk of PPVS. A small CCL/mDBLC ratio reflects the following: 1) the confluence is relatively far from the LA; and 2) there is less alignment between the confluence and the LA preoperatively. These 2 morphologic components can be impediment to perfection of direct atriovenous anastomosis. These findings provide evidence to support the previous speculation that a greater distance from the confluence to the atrial mass may increase the risk of PPVS.

Compared with prePVO, integration of the 2 3D CT modeled morphologic parameters improves risk prediction for PPVS. There are several explanations. First, prePVO consists of external expression in an anomalous connector vein, hypoplastic confluence or PVs, and functional obstruction attributed to restrictive ASD. In reality, restrictive ASD or external compression influences the procedural complexity less and can be relieved with a benign prognosis, whereas venous size is potentially the important factor as a technical issue. More importantly, the confluence features an inherent variation in not only size but also shape and orientation, thus introducing patient-specific particularities, which cannot be completely represented by prePVO. Second, an echocardiogram is an important imaging metric for assessment of prePVO, but the standard definition remains elusive, with velocity thresholds varying from 1.2 to 2 m/s among different centers.^28^ The angle of interrogation and decreased blood flow attributed to stenosis may cause inconsistence between Doppler velocity and the severity of obstruction, thereby miscategorizing patients at risk of PPVS.

Our findings may produce clinically relevant benefits in optimizing care management for this subgroup of patients in terms of assisting surgical decision making and tailoring postsurgical surveillance. The quantitative descriptors of the confluence and the LA, as well as their spatial relationships, help surgeons better comprehend patient-specific anatomy. For patients with a small iTVLC and/or CCL/mDBLC ratio, technical modifications (ie, L-shaped incision with patch augmentation,^29^ U-shaped flap technique,^30^ or window surgery^31^) can be used to achieve better alignment between the LA and the PVC as well as a large anastomosis. Additionally, increased predictive ability for PPVS by using the advanced imaging metrics can help to establish an individualized follow-up strategy. More regimented postoperative surveillance should be required for patients at higher risk for PPVS to ensure anticipatory intervention or targeted chemotherapeutic treatment in early disease to avoid aggressive propagation to upstream PV segments where treatment efficacy is limited.^9^

Several aspects warrant additional clarification. First, the higher-velocity threshold (1.8 m/s) used for diagnosis of obstruction may result in an underestimation of PPVS because lower velocities (eg, >1.5 m/s^6^ or 1.6 m/s^32^) have been used in earlier studies. Conversely, it is difficult to determine the precise velocity threshold above which stenosis develops. More importantly, echocardiographic diagnosis of stenosis should include both nonphasic flow and increased velocity. Interpreting velocity alone can be difficult because a complex interplay between velocity and severity of stenosis may contribute to the different velocity thresholds in the different settings. Second, there was an overall higher incidence of PPVS (29%) than previously reported. Plausibly, this series tends to include older patients, whereas most patients in Western countries undergo operation during the neonatal period. Previous pathologic studies^33^^,^^34^ demonstrated preoperative intrinsic hypertrophy and fibrosis of the PVs in TAPVC; longer exposure to preoperative obstruction probably results in more severe micro-obstruction of the veins. Such preoperative intrinsic abnormality of the veins may lead to hyperreactivity in the pulmonary vasculature and increase vulnerability to PPVS development.

### Study limitations

The findings of this study should be interpreted in the context of several limitations. The most prominent among these are the size and scope of the study cohort. This modestly sized investigation makes the findings exploratory, and generalizability of the study models remains unsettled, although the findings from the derivation subcohort have been externally validated in an independent subcohort from 2 other centers. Moreover, the relatively low number of PPVS events may result in reduced power, with an increased risk for a type II error. Future confirmatory studies on this topic should strive to enroll adequate numbers of cases. Additionally, although our workflow is relatively time saving (segmentation time of <5 minutes, running time of algorithm in generating quantitative geometric features of <5 minutes), the small sample size limits the ability to develop automatic segmentation of the LA and confluence. Therefore, manual reconfirmation or revision is still required to ensure the accuracy of segmentation, and this step potentially introduces intraobserver or interobserver variability. Further research is warranted to optimize the segmentation algorithm. Second, given the morphologic intricacy in these patients, other unmeasured morphologic variables may influence the results. For instance, patients with individual PV hypoplasia have a high likelihood of PPVS despite a large iTVLC or CCL/mDBLC ratio. Third, not all patients with sTAPVC during the study period were assessed, given the absence of preoperative CT scans or adequate image quality; accordingly, we cannot exclude a selection bias. Finally, because this is an observational study, we are unable to identify causative mechanisms, and as such, the results should be seen as hypothesis generating.

## Conclusions

Quantification of the confluence-atrial morphology by mathematical calculation on the basis of advanced 3D CTA modeling is potentially feasible and may optimize risk stratification. For patients with sTAPVC, the iTVLC and the CCL/mDBLC ratio are 2 useful imaging metrics for PPVS prediction.

Perspectives **COMPETENCY IN MEDICAL KNOWLEDGE:** Emerging evidence shows a relationship between the preoperative confluence-atrial morphology and PPVS in patients with TAPVC, thus creating an unmet need to characterize the confluence-atrial morphologic features more clearly. Because of the tremendous morphologic heterogeneity in these patients, single 2D size assessment cannot fully describe the confluence-atrial morphology. Our study suggests that quantifying the geometric features of the LA and PVC as well as the positional relationship between the 2 anatomical structures after accurate 3D CT modeling enhances risk evaluation in the setting of sTAPVC, and it appears to be a deeper and better metric than preoperative PVO alone to predict PPVS. Moreover, the study potentially provides implications for the clinical utility of morphologic quantification through advanced 3D CTA modeling in individualized patient care.

**TRANSLATIONAL OUTLOOK:** Future prospective, large-scale validation studies are needed to confirm the value of presurgical quantification of the confluence-atrial morphology with advanced CTA imaging in patient-specific risk stratification and to evaluate the clinical relevance of surgical strategy selection on the basis of these morphologic parameters.

## Funding Support and Author Disclosures

Support for this study has been received from the Chinese National Natural Science Foundation of China (grant Nos. 81801777, 82170307, and 81970267), Shanghai Rising-Star Program (grant No. 22QA1405800), Interdisciplinary Program of Shanghai Jiao Tong University (grant Nos. YG2022QN094, ZH2018ZDA24), Program of Shanghai Shen-kang Hospital Development Centre (grant No. SHDC2022CRD014), Shanghai Jiao Tong University Trans-med Awards Research (grant No. 20220101), and Program of Science and Technology Commission of Shanghai Municipality (grant Nos. 19411964000, 20025800300). The authors have reported that they have no relationships relevant to the contents of this paper to disclose.
