# Supplementary material for: Quantification of 3-Dimensional Confluence-Atrial Morphology in Supracardiac Total Anomalous Pulmonary Venous Connection
Source: JACC Asia. 2024 Jun 25;4(8):594–606. doi: 10.1016/j.jacasi.2024.05.002 (PMC11328765; doi:10.1016/j.jacasi.2024.05.002)
Supplement: Supplemental Tables 1 and 2 and Supplemental Figure 1 [file mmc1.docx]

**Quantification of Three-Dimensional Confluence-Atrial Morphology in Supracardiac Total Anomalous Pulmonary Venous Connection**

Guocheng Shi, MD, PhD,^a,†^ Meiping Huang,^b, †^ Yuchen Pei, PhD,^c,^ Peng Huang, MD,^d^ Chen Wen, PhD,^a^ Jin Shentu, PhD,^a^ Hao Zhang, MD, PhD,^a^ Zhongqun Zhu, MD, PhD,^a^ Yumin Zhong, MD,^e^ Lisheng Wang MD, PhD,^c,^* Huiwen Chen, MD, PhD^a,^*

**SUPPLEMENTAL MATERIALS**

**Supplemental Figure 1 Time-dependent ROC Analysis of PPVS in the Entire Cohort.** Time-dependent area under the receiver operating characteristic curve (AUC) analysis of the imaging metrics (iTVLC, CCL-to-mDBLC ratio, and integration of these 2 parameters) and the clinical risk factor (prePVO) in PPVS prediction. AUC= area under the receiver-operating characteristic; iTVLC= BSA-adjusted total volume of the left atrium and confluence; CCL= corresponding confluence length; DBLC= distance between the LA and the confluence; mDBLC= mean DBLC; prePVO= preoperative pulmonary venous obstruction; PPVS= postsurgical pulmonary vein stenosis.

**
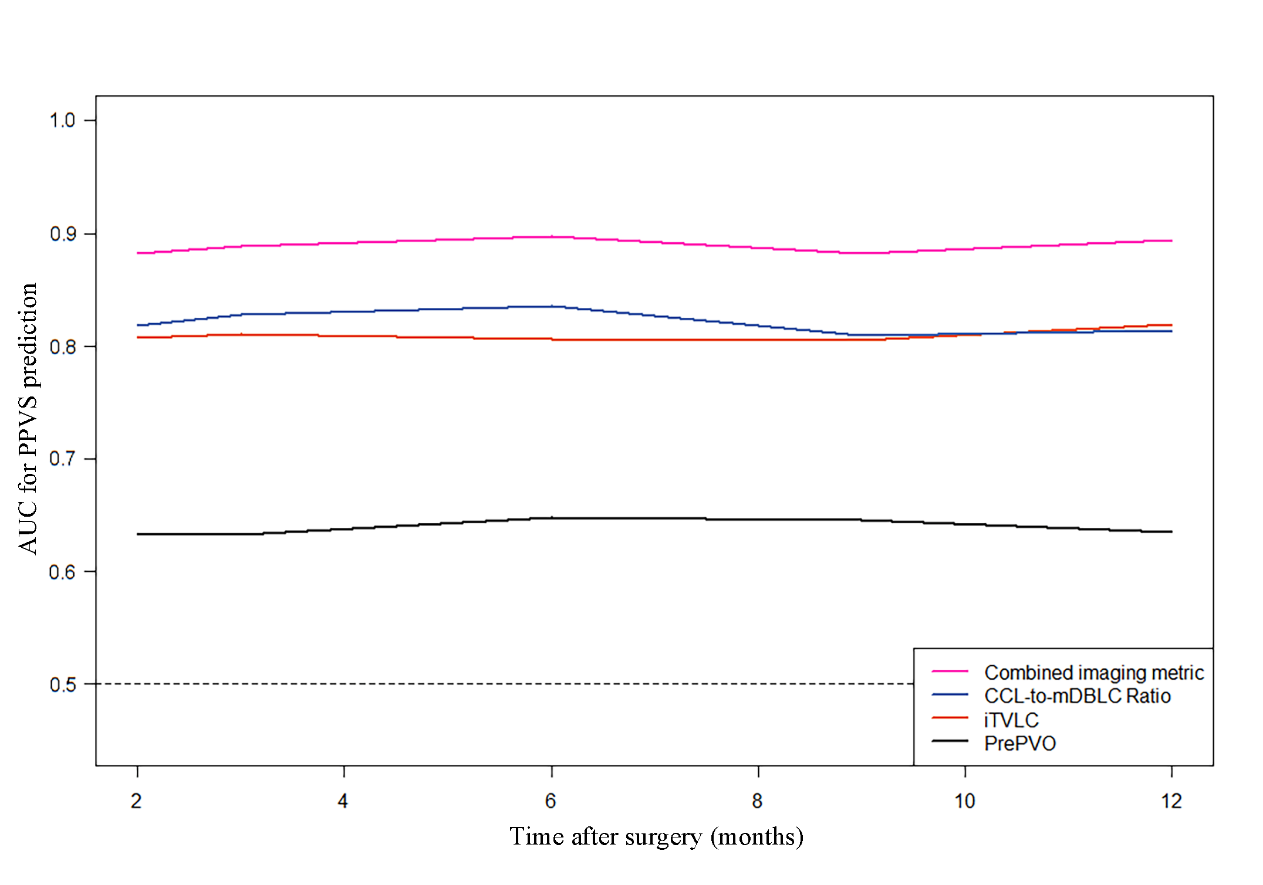
**

| **SUPPLEMENTAL TABLE 1 Comparisons of Morphological Parameters among Patients Stratified by Age** | | | | |
| --- | --- | --- | --- | --- |
| **CTA-derived Morphological Parameter** | **Neonates**  **(n=36)** | **Infants**  **(n=110)** | **Children**  **(n=16)** | ***p* Value** |
| PVC_volume_, cm^3^/m^2^ | 0.6 (0.4-0.9) | 1.3 (0.8-2.1) | 9.8 (6.0-13.9) | <0.001 |
| LA_volume_, cm^3^/m^2^ | 2.4 (1.7-3.2) | 3.9 (3.0-5.0) | 19.3 (11.5-49.7) | <0.001 |
| TVLC, cm^3^/m^2^ | 3.1 (2.3-4.0) | 5.2 (4.2-7.1) | 27.0(19.8-65.3) | <0.001 |
| iPVC_volume_, cm^3^/m^2^ | 3.1 (1.9-4.4) | 5.0 (3.6-6.9) | 11.5 (9.0-13.1) | <0.001 |
| iLA_volume_, cm^3^/m^2^ | 11.5 (10.2-15.1) | 14.5 (12.0-17.6) | 28.5 (24.2-38.5) | <0.001 |
| iTVLC, cm^3^/m^2^ | 15.8 (12.6-18.8) | 20.3 (16.5-24.2) | 42.1 (33.9-48.8) | <0.001 |
| CCL, mm | 10.1 (8.0-12.5) | 11.4 (7.9-16.5) | 14.5 (11.7-18.4) | 0.009 |
| mDBLC, mm | 1.4 (1.0-1.8) | 1.5 (1.3-1.8) | 1.7 (1.4-2.0) | 0.042 |
| CCL-to-mDBLC ratio | 7.0 (5.3-10.8) | 7.8 (4.8-13.5) | 9.2 (6.7-10.9) | 0.586 |
| CTA: computed tomography angiography; LA: left atrium; iLA_volume_: volume of LA after indexing to body surface area; iPVC_volume_: volume of pulmonary venous confluence after indexing to body surface area ; CCL: corresponding confluence length; DBLC: distance between the LA and confluence; mDBLC: mean DBLC. | | | | |

| **SUPPLEMENTAL TABLE 2 Associations Between the CTA-derived Metrics and PPVS in the Validation Cohort** | | | | | | |
| --- | --- | --- | --- | --- | --- | --- |
|  | **Unadjusted** | | | **Adjusted** | | |
| **CTA-derived Morphological Parameter** | ***p* Value** | **Hazard Ratio** | **95% CI** | ***p* Value** | **Hazard Ratio** | **95% CI** |
| iTVLC, cm^3^/m^2^ | <0.001 | 1.23 | 1.12-1.36 | 0.001* | 1.21 | 1.08-1.35 |
|  |  |  |  | <0.001^†^ | 1.22 | 1.10-1.35 |
| CCL-to-mDBLC ratio | 0.002 | 1.36 | 1.11-1.60 | 0.004* | 1.32 | 1.09-1.59 |
|  |  |  |  | 0.002^†^ | 1.36 | 1.12-1.64 |
| CTA: computed tomography angiography; CI: confidence interval; LA: left atrium; iLAv_olume_: volume of LA after indexing to body surface area; iPVC_volume_: volume of pulmonary venous confluence after indexing to body surface area; CCL: corresponding confluence length; DBLC: distance between the LA and confluence; *: after adjustment for patient age;  ^†^ :after adjustment for preoperative PVO. | | | | | | |
